# COVID‐19 Vaccine Effectiveness Against Hospitalization in Older Adults, VEBIS Hospital Network, Europe, September 2024–May 2025

**DOI:** 10.1111/irv.70191

**Published:** 2025-11-25

**Authors:** Madelyn Rojas‐Castro, Nuno Verdasca, Susana Monge, Laurane De Mot, Camino Trobajo‐Sanmartín, Róisín Duffy, Gergő Túri, Monika Kuliese, Ralf Duerrwald, Maria‐Louise Borg, Odette Popovici, Verónica Gomez, Zvjezdana Lovrić Makarić, Odile Launay, Diogo F. P. Marques, Francisco Pozo, Arne Witdouck, Iván Martínez‐Baz, Margaret Fitzgerald, Beatrix Oroszi, Ligita Jančorienė, Silke Buda, Ausra Dziugyte, Mihaela Lazăr, Ausenda Machado, Irena Tabain, Liem Binh Luong Nguyen, Eva Rivas Wagner, François Dufrasne, Jesús Castilla, Lisa Domegan, Viktória Velkey, Fausta Majauskaite, Carolin Hackmann, Nathalie Nicolay, Sabrina Bacci, Angela M. C. Rose, European Hospital Vaccine Effectiveness Group

**Affiliations:** ^1^ Epiconcept Paris France; ^2^ Infectious Diseases Department National Health Institute Doutor Ricardo Jorge Lisbon Portugal; ^3^ National Centre of Epidemiology, CIBERINFEC Institute of Health Carlos III Madrid Spain; ^4^ Scientific Directorate of Epidemiology of Infectious Diseases, Sciensano Brussels Belgium; ^5^ Instituto de Salud Pública de Navarra – IdiSNA – CIBERESP Pamplona Spain; ^6^ Health Service Executive‐Health Protection Surveillance Centre (HPSC) Dublin Ireland; ^7^ National Laboratory for Health Security, Epidemiology and Surveillance Centre Semmelweis University Budapest Hungary; ^8^ Department of Infectious Diseases Lithuanian University of Health Sciences Kaunas Lithuania; ^9^ National Reference Centre for Influenza Robert Koch Institute Berlin Germany; ^10^ Infectious Disease Prevention and Control Unit (IDCU) Msida Malta; ^11^ National Institute of Public Health (INSP/NIPH) ‐ National Centre for Surveillance and Control of Communicable Diseases Bucharest Romania; ^12^ Department of Epidemiology National Institute of Health Doctor Ricardo Jorge Lisbon Portugal; ^13^ Divison for Epidemiology of Communicable Diseases Croatian Institute of Public Health Zagreb Croatia; ^14^ INSERM CIC 1417, F‐CRIN, I‐REIVAC Network, Assistance Publique‐Hôpitaux de Paris Hôpital Cochin Paris Paris France; ^15^ National Centre of Microbiology, CIBERESP Institute of Health Carlos III Madrid Spain; ^16^ Department of Internal Medicine and Infectious Diseases Universitair Ziekenhuis Brussel Brussels Belgium; ^17^ Clinic of Infectious Diseases and Dermatovenerology, Institute of Clinical Medicine, Medical Faculty Vilnius University Vilnius Lithuania; ^18^ National Institute of Research & Development for Microbiology & Immunology (INCDMM) “Cantacuzino” Bucharest Romania; ^19^ Department for Direct Diagnostic Virology Croatian Institute of Public Health Zagreb Croatia; ^20^ Unida de Vigilancia Epidemiológica de la Dirección General de Salud Pública, Servicio Canario de la Salud Islas Canarias Spain; ^21^ National Reference Center for Respiratory Pathogens, Belgium, Sciensano Brussels Belgium; ^22^ Department of Infectious Disease Epidemiology, Respiratory Infections Unit Robert Koch Institute Berlin Germany; ^23^ European Centre for Disease Prevention and Control Stockholm Sweden

**Keywords:** case–control study, elderly, severe acute respiratory infection (SARI), test‐negative design, vaccine effectiveness

## Abstract

We estimated COVID‐19 vaccine effectiveness (VE) against PCR‐confirmed SARS‐CoV‐2 hospitalization in patients ≥ 60 years with severe acute respiratory infection, using a multicenter, test‐negative, case–control study across seven sites in six European countries between September 2024 and May 2025. We included 352 cases (115 vaccinated; 33%) and 9980 controls (5024 vaccinated; 50%). VE was 42% (95% CI: 15; 61) 14–59 days post‐vaccination, 32% (95% CI: −1; 54) at 60–119 days, and 36% (95% CI: 2; 60) at 120–179 days, and no effect thereafter. Among adults aged 60–79 and ≥ 80 years, we observed moderate VE against COVID‐19 hospitalization for up to 2 and 4 months, respectively.

## 1 Introduction

Between September and October 2024, COVID‐19 vaccination campaigns were launched in many European Union/European Economic Area (EU/EEA) countries, mainly targeting adults over 60 or 65 years old and other high‐risk individuals. In the EU/EEA, the Comirnaty Omicron JN.1 vaccine was the primary vaccine administered in EU/EEA countries, with low to moderate COVID‐19 vaccination coverage and wide country variation (7% overall, range < 1%–53%) [1, 2, 3].

From the start of the autumn/winter 2024/25 season, SARS‐CoV‐2 positivity rates decreased, following elevated activity during the summer 2024 [4, 5]. The KP.3 lineage of the Omicron BA.2.86 variant and XEC—a recombinant lineage of the Omicron BA.2.86 (KS.1.1 and KP.3.3)—co‐circulated, followed by an increase in the LP.8.1 lineage [5]. Monitoring COVID‐19 vaccine effectiveness (VE) overall and over time is essential, as older adults remain at higher risk of severe outcomes [1], and waning of vaccines has been reported [6].

Our aim was to provide COVID‐19 VE estimates, overall and by time since vaccination (TSV), against PCR‐confirmed SARS‐CoV‐2 hospitalization in Europe from September 19, 2024 to May 4, 2025 among patients with severe acute respiratory infection (SARI) aged ≥ 60 years.

## 2 VEBIS Hospital VE Network

**FIGURE 1:**
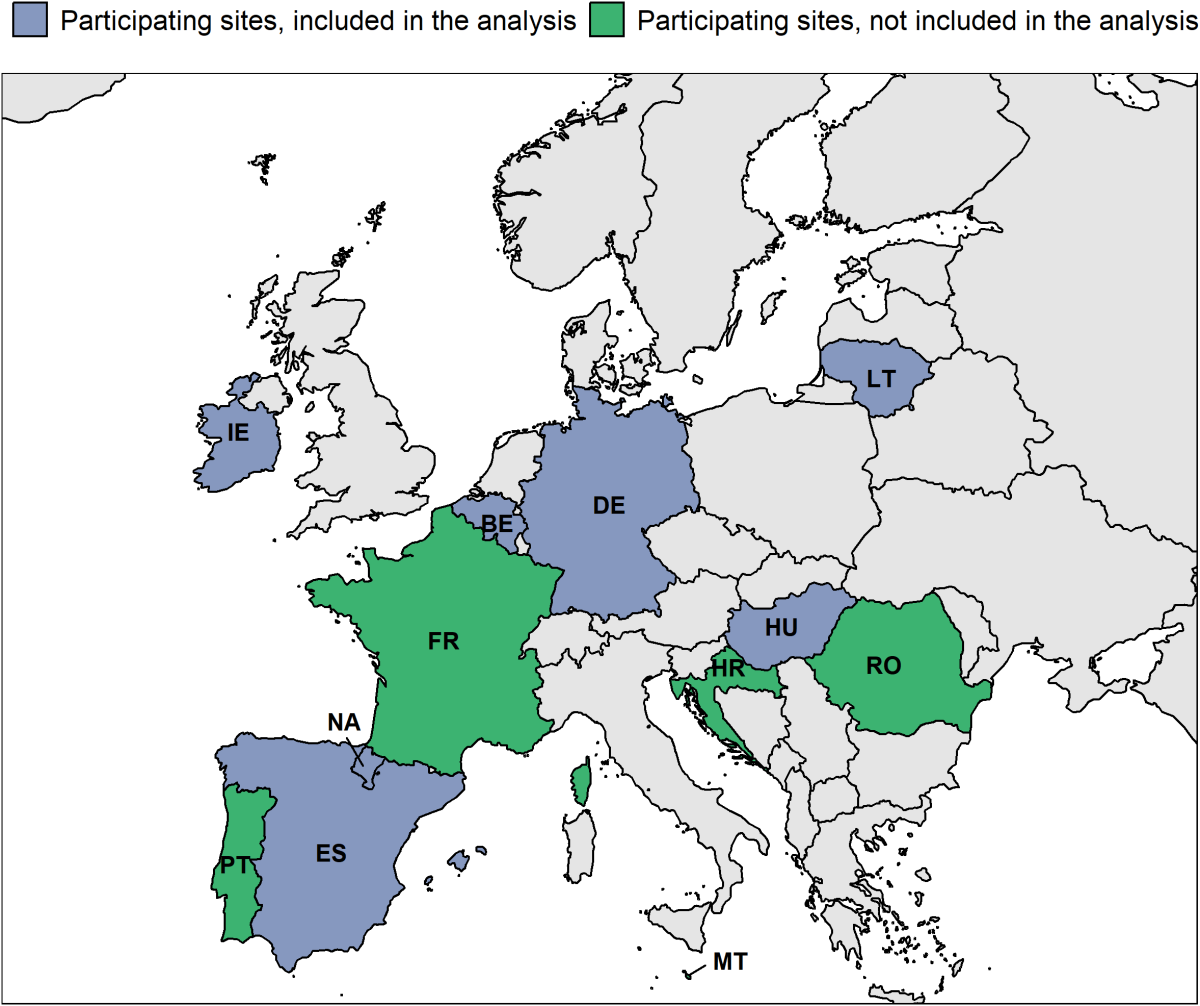
Countries and study sites^a^ included in the VEBIS hospital network and in the analysis, Europe, September 29, 2024–May 4, 2025. VEBIS: Vaccine Effectiveness, Burden and Impact Studies. (a) Twelve participating sites: Belgium (BE), Croatia (HR), France (FR), Germany (DE), Hungary (HU), Ireland (IE), Lithuania (LT), Malta (MT), Navarre region Spain (NA), Portugal (PT), Spain (ES), and Romania (RO). Sites included in analysis: BE, DE, HU, IE, LT, NA, and ES.

This multicenter, test‐negative, case–control hospital‐based study is part of the Vaccine Effectiveness, Burden and Impact Studies (VEBIS) project. VEBIS includes 100 hospitals across 11 European countries (Figure 1), following a common generic protocol [6].

## 3 Definitions and Analysis Restrictions

We defined patients with SARI as those hospitalized for ≥ 24 h with at least one symptom (out of fever, cough, or shortness of breath), and included those with symptoms occurring at least 14 days after the start of the autumn 2024/25 vaccination campaign in their country and until the date of onset of symptoms of the last positive case before May 4, 2025 (with some country differences; Table S1). We defined cases as patients with SARI testing positive for SARS‐CoV‐2 by RT‐PCR within 48 h of admission, and controls as those testing PCR‐negative, with no positive test in the previous 14 days [7].

We classified a patient as vaccinated if they received a vaccine ≥ 14 days before symptom onset during the country's autumn 2024 campaign; otherwise, they were unvaccinated. We excluded those vaccinated 1–13 days before symptom onset and those (vaccinated or unvaccinated) who had received a vaccine in the180 days preceding the campaign (where known). We also excluded (where known) all patients vaccinated with vaccines other than those recommended by the European Medicines Agency (EMA).

We restricted our analysis to patients aged ≥ 60 years who were targeted to receive a COVID‐19 vaccine in country‐specific campaigns (with some country differences; Table S1). Healthcare workers and patients living in a long‐term care facility were excluded.

We excluded patients with missing/erroneous information for variables included in the analysis (sex, age, number of chronic conditions, and date of last COVID‐19 vaccine dose), as well as sites with fewer than five cases or controls, or with no vaccinated patients in both case and control groups (Figure S1).

## 4 Statistical Methods

We estimated the odds ratio (OR) of vaccination between cases and controls using logistic regression, adjusting the OR by study site, date of symptom onset, sex, age, and number of chronic conditions. The VE was calculated as $\left(1-\text{adjusted OR}\right)\times 100\%$. Pooled VE was estimated overall and stratified by TSV using 60‐day cutoffs, for all patients aged ≥ 60, 60–79, and ≥ 80 years.

The best functional forms of the continuous variables age and onset date (categories, splines, linear terms) were selected using the Akaike information criterion. We carried out a complete case analysis.

Estimates were not shown if there were < 10 vaccinated patients, or when the VE estimate had an absolute difference > 10% from VE estimated using penalized logistic regression using Firth's method [8].

The following sensitivity analyses were performed: (a) changing the days since vaccination, and excluding (b) recently vaccinated patients, (c) influenza‐positive controls, (d) co‐infections (Table S2).

Analyses were conducted using R Version 4.4.2, with R via the “logistf” package [9].

## 5 Description of SARI Patients

After exclusions, we included 352 cases and 9980 controls from 72 hospitals in seven sites (Figure S1). A total of 115 (33%) cases and 5024 (50%) controls were vaccinated (Figure S2). The median time between vaccination and symptom onset was 98 days (interquartile range, IQR: 54–142, max: 209 days) for cases and 93 days (IQR: 61–132; max: 213 days) for controls (Table 1). The proportion of vaccinated controls among participating sites ranged from 3% (Hungary) to 60% (Navarre region) (Table S3).

**TABLE 1:** Characteristics of SARS‐CoV‐2 cases and controls, VEBIS hospital study, Europe, September 29, 2024–May 4, 2025 (*n* = 10,332).

Characteristic	SARS‐CoV‐2 cases		Controls		*p*
	(*n* = 352)		(*n* = 9980)		
	*n*	%	*n*	%	
Age (years)					
60–69	59	17	2098	21	0.147^a^
70–79	113	32	3033	30	
≥ 80	180	51	4849	49	
Median (IQR)	80 (72–87)		79 (74–87)		
Female	155	44	4859	49	0.096^a^
Any chronic condition^b^	299	85	8437	85	0.881^a^
Number of chronic condition^b^					
No conditions	53	15	1551	16	0.980^a^
One condition	71	20	2012	20	
Two or more conditions	228	65	6417	64	
Vaccination status					
Vaccinated^c^	115	33	5024	50	< 0.001^a^
Unvaccinated^d^	237	67	4956	50	
Days from the last dose to symptom onset^e^					
Median (IQR); max	98 (54–142); 209		93 (61–132); 213		
Vaccine type (where known)					
Comirnaty KP.2 (BioNTech/Pfizer)	7	6	397	8	0.856^g^
Comirnaty JN.1 (BioNTech/Pfizer)	79	69	3431	68	
Comirnaty XBB.1.5 (BioNTech/Pfizer)	0	0	12	0	
Comirnaty unspecified (BioNTech/Pfizer)^f^	28	25	1169	23	
Spikevax (Moderna)	0	0	1	0	
Any severe outcome					
Yes	25	8	977	10	0.133^a^
No	304	92	8498	90	
*Missing*	23	7	505	5	
ICU admission	9	3	520	5	0.036^a^
Death	17	5	531	6	0.842^a^
Median length of hospital stay in days (IQR)	6 (4–9)		6 (4–9)		
Presence of other respiratory pathogens					
Influenza	12	3	1818	18	< 0.001^a^
RSV	8	3	828	9	< 0.001^a^

Abbreviations: ICU: intensive care unit; IQR: interquartile range; RSV: respiratory syncytial virus; VEBIS: Vaccine Effectiveness, Burden and Impact Studies.

^a^ *p* calculated using the Chi‐square test (*p*‐value threshold for statistical significance: 0.05).

^b^ Among commonly collected chronic conditions: diabetes, heart disease, lung disease/asthma, and immunodeficiency.

^c^ Received a COVID‐19 vaccine dose during the autumn 2024 vaccination campaign in each country. Dates of start of each country's vaccination campaign are in Table S1.

^d^ Never vaccinated for COVID‐19 or with the last COVID‐19 vaccination dose received ≥ 180 days prior to the start of the 2024 vaccination campaign in each country (Table S1).

^e^ Restricted to those vaccinated with a COVID‐19 vaccine during autumn 2024 vaccination campaigns. Onset dates for all sites with > 5% missing data, using median delay to hospital admission per site, but only if there were more than 30 patients in each age group for this median estimation (60–79, ≥ 80 years). Otherwise, these patients were excluded.

^f^ Undefined Comirnaty (BioNTech/Pfizer) formulation between XBB.1.5, JN.1, and KP.2 brands available during the autumn 2024 vaccination campaigns.

^g^ *p* calculated using Fisher's exact test (*p*‐value threshold for statistical significance: 0.05).

## 6 Vaccine Effectiveness

**FIGURE 2:**
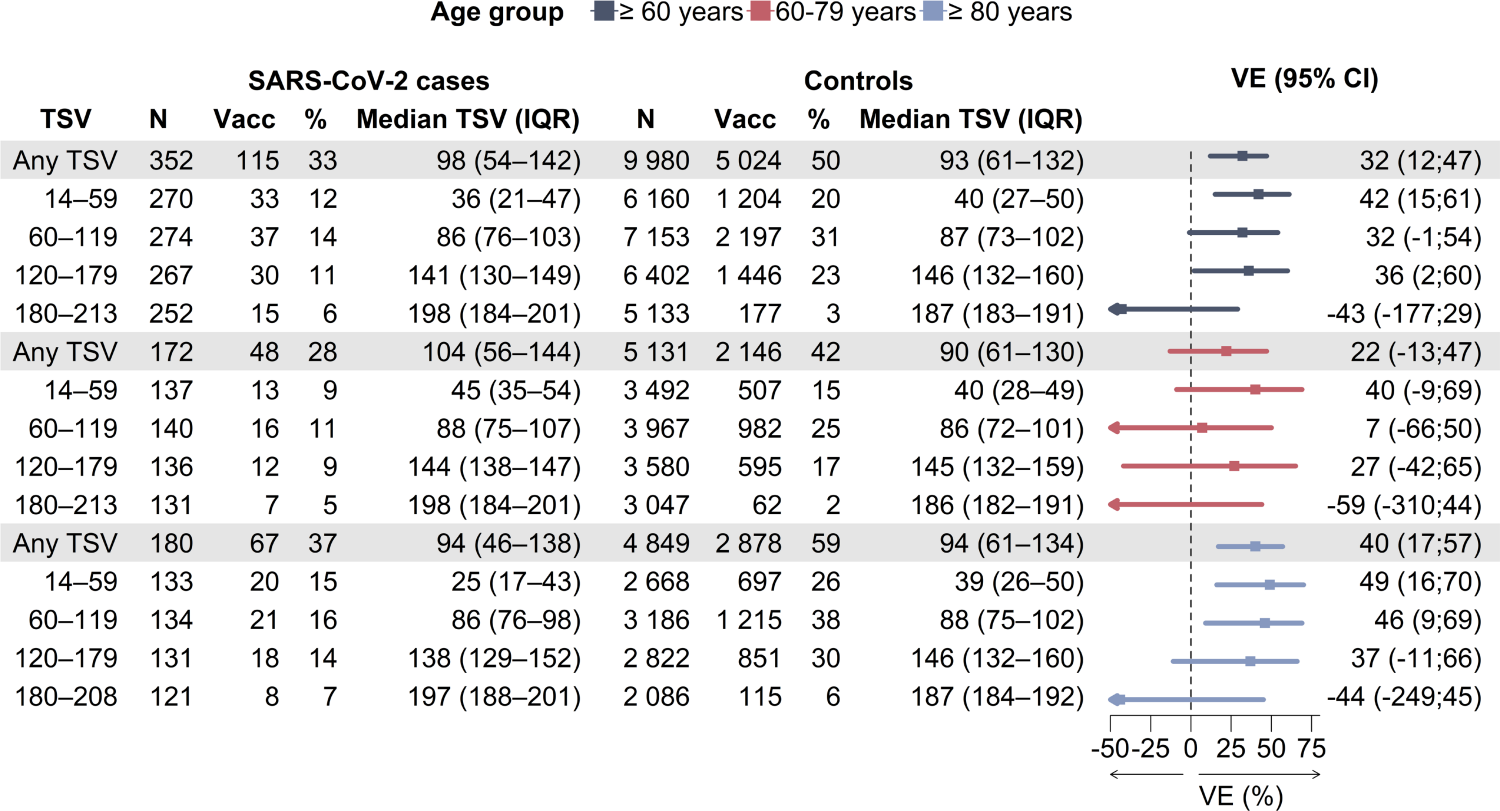
Vaccine effectiveness of COVID‐19 vaccines against hospitalization among patients with SARI, by time since vaccination (60‐day cut‐offs) and by age group (≥ 60, 60–79, and ≥ 80 years old), autumn 2024 vaccination campaign, VEBIS hospital study, Europe, September 29, 2024–May 4, 2025 (*n* = 10332). CI: confidence interval, IQR: interquartile range, TSV: time since vaccination (days from the last COVID‐19 vaccination dose to symptom onset), VE: vaccine effectiveness, grey shading: any time since vaccination.

The VE among patients with SARI aged ≥ 60 years was 42% (95% CI: 15; 61) in the first 14–59 days post‐vaccination, 32% (95% CI: −1; 54) between 60 and 119 days, and 36% (95% CI: 2; 60) at 120–179 days post‐vaccination, with no effect thereafter (Figure 2).

For patients aged 60–79 years, VE was 40% (95% CI: −9; 69) at 14–59 days, 7% (95% CI: −66; 50) at 60–119 days, and 27% (95% CI: −42; 65) at 120–179 days post‐vaccination, with no effect thereafter (Figure 2). For patients aged ≥ 80 years, VE was 40% (95% CI: 17; 57) overall (14–208 days post‐vaccination). The VE was 49% (95% CI: 16; 70) at 14–59 days, 46% (95% CI: 9; 69) at 60–119 days, 37% (95% CI: −11; 66) at 120–179 days post‐vaccination, with no effect thereafter (Figure 2). Comirnaty (BioNTech/Pfizer) JN.1 VE is presented in Figure S4.

Sensitivity analyses yielded results similar to those of the main analysis, with a maximum absolute difference in the overall VE from the main analysis of ≤ 4% in both age groups (Table S2).

## 7 Discussion

Our results suggest that, between September 29, 2024 and May 4, 2025, the autumn 2024 COVID‐19 vaccine (mainly the Comirnaty JN.1‐adapted mRNA vaccine) conferred moderate protection against co‐circulating BA.2.86 lineages (KP.3, XEC and LP.8.1) at ~42% in the first 59 days post‐vaccination for all age groups ≥ 60 years, amid low SARS‐CoV‐2 circulation and low‐medium COVID‐19 vaccination coverage in participating countries. We found sustained protection up to 2 months among those aged 60–79 years, and up to 4 months post‐vaccination among those ≥ 80 years.

This autumn/winter season VE estimates were lower than our early 2023/24 results among those aged 60–79 and ≥ 80 years, at 59% and 76% at 14–29 days, and 42% and 55% at 30–59 days post‐vaccination, respectively [8]—but were similar to those for the BA.2.86/JN.1 predominant period (47% and 45% at 14–59 days post‐vaccination), with no vaccine effect observed after 4 months in any age group [11].

Our VE estimates were similar to interim estimates from two US studies using EHR‐based test‐negative and cohort designs in adults ≥ 65 years, reporting 45% and 46% at 14–119 days post‐vaccination in the VISION/IVY networks [12], and 46% in insured patients in two US states [13]. Other studies reported higher VE: a test‐negative case–control study in US veterans [14] estimated 75% VE for the adapted KP.2 COVID‐19 vaccine; three interim studies in EU/EEA found VE ranging from 60% to 70%: The id. Drive platform reported 68% for Spikevax JN.1 (Moderna) in adults ≥ 18 years across three countries [15], the VEBIS EHR‐based cohort study reported 60% across six countries [16], and a Danish nationwide study (included in the VEBIS EHR network) reported 70% VE with protection sustained up to 4 months [17]. To our knowledge, to date, our study is the only one reporting both waning of vaccines and differential waning by age during the 2024/25 season.

The moderate VE in our study may be related to an increased population immunity after high summer 2024 incidence [3, 4, 5], as differential prior infection between vaccinated and unvaccinated groups may play a role; however, this information was not available. The predominant use of the adapted COVID‐19 JN.1 vaccine is not expected to play a significant role, as both JN.1 and KP.2 vaccines induce robust protection against JN.1 subvariants, although some degree of immune escape is expected against KP.3.1.1, XEC, and LP.8.1 [18, 19, 20]. However, differences in subvariant co‐circulation across countries, population characteristics, reasons for hospitalization, and adaptations in the SARI case definition may play a role. Random variation cannot be ruled out, as well as study design differences (e.g., test‐negative versus cohort accounting differently for health‐seeking behavior [13, 14]), potential biases, such as depletion of susceptibles, residual confounding from unmeasured reasons for vaccination (e.g., doses number, degree of fragility), and healthy vaccine effect or frailty bias. Differential waning this season may reflect age‐related differences in baseline risk or vaccination timing not fully captured in our study.

Our analysis was limited by sample size, due to low circulation of SARS‐CoV‐2. Estimates and trends by TSV should be interpreted with caution as precision was low, and confidence intervals overlapped. Even so, all estimates presented here fulfilled criteria established a priori to minimize small sample bias. Sequencing data were limited and precluded robust sublineage‐specific VE estimates, but indicated a signal of similar VE against the two dominant lineages sequenced (KP.3 vs. XEC) (data not shown). Other limitations were similar to those described previously [10, 11].

The strengths of our study include its multicenter component, with a generic protocol to mitigate potential sources of heterogeneity and increase internal validity. This, and a well‐established hospital network, enables us to provide timely pooled post‐marketing, independent estimates of seasonal COVID‐19 VE in Europe.

## 8 Conclusion

Despite low to moderate vaccination rates, we show that in our participating sites, COVID‐19 vaccines provided moderate, but short‐lived protection against COVID‐19 hospitalization during autumn/winter 2024–25, particularly for those aged 60–79 years.

## Author Contributions

**Madelyn Rojas‐Castro:** data curation (lead), formal analysis (lead), investigation (equal), methodology (lead), software (lead), Data Curation (equal); validation (equal), visualization (lead), writing – original draft (lead), writing – review and editing (lead).**Nuno Verdasca:** formal analysis, investigation, methodology, software, validation, writing – review and editing. **Susana Monge:** writing – review and editing, data curation, formal analysis, investigation, methodology, resources. **Laurane De Mot:** writing – review and editing, resources, methodology, investigation, formal analysis, data curation. **Camino Trobajo‐Sanmartín:** investigation, methodology, writing – review and editing, formal analysis, data curation, resources. **Róisín Duffy:** investigation, methodology, writing – review and editing, formal analysis, data curation, resources. **Gergő Túri:** investigation, methodology, writing – review and editing, formal analysis, data curation, resources. **Monika Kuliese:** investigation, methodology, writing – review and editing, formal analysis, data curation, resources. **Ralf Duerrwald:** data curation (lead), investigation (equal), resources (equal), supervision (equal), writing – review and Editing (equal). **Maria‐Louise Borg:** investigation, methodology, writing – review and editing, formal analysis, data curation, resources. **Odette Popovici:** investigation, methodology, writing – review and editing, formal analysis, data curation, resources. **Verónica Gomez:** data curation (lead), investigation (equal), resources (equal), supervision (equal), writing – review and Editing (equal).**Odile Launay:** investigation, methodology, writing – review and editing, formal analysis, data curation, resources. **Diogo F. P. Marques:** data curation (lead), software (equal), validation (lead), writing – review and editing (equal). **Francisco Pozo:** investigation, methodology, writing – review and editing, formal analysis, data curation, resources. **Arne Witdouck:** investigation, methodology, writing – review and editing, formal analysis, data curation, resources. **Iván Martínez‐Baz:** investigation, methodology, writing – review and editing, formal analysis, data curation, resources. **Margaret Fitzgerald:** investigation, methodology, writing – review and editing, formal analysis, data curation, resources. **Beatrix Oroszi:** investigation, methodology, writing – review and editing, formal analysis, data curation, resources. **Silke Buda:** investigation, methodology, writing – review and editing, formal analysis, data curation, resources. **Ligita Jan?orien?:** data curation (lead), investigation (equal), resources (equal), supervision (equal), writing – review and Editing (equal). **Ausra Dziugyte:** investigation, methodology, writing – review and editing, formal analysis, data curation, resources. **Mihaela Laz?r:** data curation (lead), investigation (equal), resources (equal), supervision (equal), writing – review and Editing (equal).**Ausenda Machado:** investigation, methodology, writing – review and editing, formal analysis, data curation, resources. **Irena Tabain:** investigation, methodology, writing – review and editing, formal analysis, data curation, resources. **Liem Binh Luong Nguyen:** data curation (lead), investigation (equal), resources (equal), supervision (equal), writing – review and Editing (equal).**François Dufrasne:** investigation, methodology, writing – review and editing, formal analysis, data curation, resources. **Jesús Castilla:** investigation, methodology, writing – review and editing, formal analysis, data curation, resources. **Lisa Domegan:** investigation, methodology, writing – review and editing, formal analysis, data curation, resources. **Viktória Velkey:** investigation, methodology, writing – review and editing, formal analysis, data curation, resources. **Fausta Majauskaite:** investigation, methodology, writing – review and editing, formal analysis, data curation, resources. **Carolin Hackmann:** investigation, methodology, writing – review and editing, formal analysis, data curation, resources. **Nathalie Nicolay:** conceptualization, investigation, writing – review and editing, funding acquisition, project administration. **Sabrina Bacci:** conceptualization, investigation, writing – review and editing, funding acquisition, project administration. **Angela M. C. Rose:** conceptualization (equal), project administration (lead), resources (lead), supervision (lead), methodology (supporting), visualization (supporting), writing – review and Editing (equal).**European Hospital Vaccine Effectiveness Group:** investigation, methodology, writing – review and editing, formal analysis, data curation, resources.

## Ethics Statement

The planning, conducting and reporting of the studies were in line with the Declaration of Helsinki. Official ethical approval was not required if studies were classified as being part of routine care/surveillance (Ireland, Spain); in Belgium and Germany, VE estimation is included in SARI surveillance. Verbal informed consent, however, is required from patients for participation in any further research (including VE studies). Other study sites received local ethical approval from a national or regional review board: in Belgium, the study protocol was approved by the central Ethical Committee (CHU Saint‐ Pierre [AK/12‐02‐11/4111] initially in 2011 and UZ VUB [B.U.N. 143,201,215,671] from 2014 on) and each participating hospital's local ethical committees. The most recent amendment was approved on 27/9/2023 (reference 2012/310 Am6). The German SARI surveillance was approved by the Charité‐ Universitätsmedizin Berlin Ethical Board (Reference EA2/126/11 and EA2/218/19). The Lithuania study was approved on July 3, 2020 by the Lithuanian Biomedical Research Ethics Committee No.: L‐20‐3/1; and later permission was extended for the study period for seasons 2020–2025. The Hungary study protocol was approved by the National Scientific and Ethical Committee (IV/1885‐5/2021/EKU), and the most recent amendment was approved on 4/10/2024 (BM/25007‐2/2024).

## Consent

Written informed consent for participation and publication of data was obtained from all participants in accordance with ethical guidelines.

## Conflicts of Interest

Ligita Jančorienė has received honoraria fees for lectures from Pfizer, Viatris, Swixx Biopharma. All other authors declare no conflicts of interest.

## Supporting information


**Table S1:** Summary of the 2024/25 vaccination campaign target, vaccines, and period in analysis by site in the VEBIS hospital study, September 29, 2024–May 4, 2025
**Table S2:** (Sensitivity analyses vaccine effectiveness of COVID‐19 vaccines against hospitalization among patients with SARI any time since vaccination), autumn 2024 vaccination campaign, VEBIS hospital study, Europe, September 29, 2024–May 4, 2025
**Table S3:** Comparison of the proportion of vaccinated controls by analysis site vs. national COVID‐19 vaccine coverage
**Figure S1:** Patient exclusion flowchart, VEBIS hospital study, September 19, 2024–May 4, 2025
**Figure S2:** Number of cases and controls by week of symptoms onset, VEBIS hospital study, Europe, September 29, 2024–May 4, 2025 (*n* = 10,332)
**Figure S3:** (A) Number of COVID‐19 cases by week of symptom onset by sublineage/lineage, B) Proportion of COVID‐19 cases sequenced by week of symptoms, VEBIS hospital study, Europe, September 29, 2024–May 4, 2024
**Figure S4:** Vaccine effectiveness of Comirnaty JN.1 vaccine against hospitalization among patients with SARI, by time since vaccination (60‐day cut‐offs), autumn 2024 vaccination campaign, VEBIS hospital study, Europe, September 29, 2024–May 4, 2025 (*n* = 5740)

## Data Availability

Data are available on request.
