# Supplementary material for: COVID‐19 Vaccine Effectiveness Against Hospitalization in Older Adults, VEBIS Hospital Network, Europe, September 2024–May 2025
Source: Influenza Other Respir Viruses. 2025 Nov 25;19(11):e70191. doi: 10.1111/irv.70191 (PMC12646827; doi:10.1111/irv.70191)
Supplement: Supplementary file 1 — Table S1: Summary of the 2024/25 vaccination campaign target, vaccines, and period in analysis by site in the VEBIS hospital study, September 29, 2024–May 4, 2025 Table S2: (Sensitivity analyses vaccine effectiveness of COVID‐19 vaccines against hospitalization among patients with SARI any time since vaccination), autumn 2024 vaccination campaign, VEBIS hospital study, Europe, September 29, 2024–May 4, 2025 Table S3: Comparison of the proportion of vaccinated controls by analysis site vs. national COVID‐19 vaccine coverage Figure S1: Patient exclusion flowchart, VEBIS hospital study, September 19, 2024–May 4, 2025 Figure S2: Number of cases and controls by week of symptoms onset, VEBIS hospital study, Europe, September 29, 2024–May 4, 2025 (n = 10,332) Figure S3: (A) Number of COVID‐19 cases by week of symptom onset by sublineage/lineage, B) Proportion of COVID‐19 cases sequenced by week of symptoms, VEBIS hospital study, Europe, September 29, 2024–May 4, 2024 Figure S4: Vaccine effectiveness of Comirnaty JN.1 vaccine against hospitalization among patients with SARI, by time since vaccination (60‐day cut‐offs), autumn 2024 vaccination campaign, VEBIS hospital study, Europe, September 29, 2024–May 4, 2025 (n = 5740) [file IRV-19-e70191-s001.docx]

# Supplementary material

#### Table S1. Summary of the 2024/25 vaccination campaign target, vaccines, and period in analysis by site in the VEBIS hospital study, 29 September 2024–4 May 2025

| **Site** | **Age (target population)^a^** | **Available COVID-19 vaccine brands in the 2024/2025 vaccination campaign** | **National date of start of vaccination campaign** | **VEBIS hospital study**  **Date of onset of symptoms of the last positive case before 4 May 2025** |
| --- | --- | --- | --- | --- |
| Belgium | 65y^b^ | Comirnaty JN.1/KP.2 | 15 Sep 2024 | 30 Apr 2025 |
| Germany | 60y | Comirnaty JN.1/KP.2, Spikevax, Nuvaxovid | 01 Oct 2024 | 15 Apr 2025 |
| Hungary | 60y | Spikevax JN.1 | 02 Dec 2024 | 01 Mar 2025 |
| Ireland | 60y | Comirnaty JN.1 | 30 Sep 2024 | 23 Mar 2025^c^ |
| Lithuania | All ages | Comirnaty JN.1 | 17 Sep 2024 | 14 Feb 2025 |
| Spain | 60y | Comirnaty JN.1/KP.2 | 23 Sep 2024–23 Oct 2024^d^ | 03 May 2025 |

VEBIS: Vaccine Effectiveness, Burden and Impact Studies.

^a^ Recommended vaccination age regardless of chronic disease in autumn vaccination campaigns in each country. Other chronic conditions might have been included in the vaccination target group, but information on these was not collected in the study.

^b^ In Belgium, due to age-specific recommendations, all those ≥65 years were included and only those with chronic conditions aged 60–64 years (asthma; cancer; dementia; diabetes; heart disease; immunodeficiencies; liver disease; lung disease; neurological disease; neuromuscular disorder; obesity; renal disease).

^c^ In Ireland, the study period ended with the onset of their spring 2025 campaign in March.

^d^ In Spain, we consider different start of vaccination campaign by region; in the table the earliest and the latest dates of the start of the vaccination campaign by region are shown

**Table S2. Sensitivity Analyses vaccine effectiveness of COVID-19 vaccines against hospitalisation among SARI patients any time since vaccination), autumn 2024 vaccination campaign, VEBIS hospital study, Europe, 29 September 2024–4 May 2025**

| **Sensitivity Analysis** | **Seasonal COVID-19 vaccination** | **SARS-CoV-2 cases** | | **Controls** | | **VE (95% CI)** | **Absolute**  **Difference (%)** |
| --- | --- | --- | --- | --- | --- | --- | --- |
|  |  | **N cases** | **Median TSV (IQR)** | **N controls** | **Median TSV (IQR)** |  |  |
| **1)** **Days since vaccination:** use 7 instead of 14 days for defining vaccinated patients and study start date | Unvaccinated | 274 |  | 5 114 |  | Ref. |  |
|  | Vaccinated | 121 | 90 (57–131) | 5 163 | 91 (47–141) | 33 (15;47) | 1% |
| **2.1) Recent vaccination:** Retain patients vaccinated ≤180 days before the start of the vaccination campaign | Unvaccinated | 242 |  | 5 008 |  | Ref. |  |
|  | Vaccinated | 115 | 98 (53–142) | 5 033 | 92 (61–132) | 32 (14;47) | <1% |
| **2.2) Recent vaccination:**  Exclude patients with the last dose at 90 instead of 180 days | Unvaccinated | 240 |  | 4 983 |  | Ref. |  |
|  | Vaccinated | 115 | 98 (53–142) | 5 033 | 92 (61–132) | 32 (13;47) | <1% |
| **2.3) Recent vaccination:**  Exclude patients with the last dose at 270 days instead of 180 days | Unvaccinated | 236 |  | 4 876 |  | Ref. |  |
|  | Vaccinated | 114 | 96 (53–143) | 4 904 | 93 (61–132) | 32 (13;47) | <1% |
| **3) Influenza positive controls:** Exclude influenza-positive controls | Unvaccinated | 237 |  | 4 004 |  | Ref. |  |
|  | Vaccinated | 115 | 98 (53–142) | 4 158 | 94 (58–137) | 34 (15;49) | 2% |
| **4) Co-infections:** Exclude cases with known influenza or RSV co-infection | Unvaccinated | 227 |  | 4 956 |  | Ref. |  |
|  | Vaccinated | 106 | 102 (54–144) | 5 024 | 93 (61–132) | 32 (12;48) | <1% |

#### CI: confidence interval, IQR: interquartile range, TSV: time since vaccination (days from the last COVID-19 vaccination dose to symptom onset), VE: vaccine effectiveness

#### Table S3. Comparison of the proportion of vaccinated controls by analysis site vs national COVID-19 vaccine coverage

| **Site** | **VEBIS-hospital study**  **% vaccinated controls**  **(**September 2024–May 2025) | | | | | **National COVID-19 vaccine coverage (≥60y)**  (August 2024 – January 2025 )^a^ | | | |
| --- | --- | --- | --- | --- | --- | --- | --- | --- | --- |
|  | **≥60y** | **60–69y** | **70–79y** | | **≥80y** | **60–69y** | **70–79y** | | **≥80y** |
| Belgium | 47% | 38% | 44% | 55% | | 12% | 43% | 47% | |
| Germany | 25% | 38% | 35% | 10% | | NDR | NDR | NDR | |
| Hungary | 3% | 7% | 2% | 0% | | <1% | <1% | <1% | |
| Ireland | 46% | 29% | 52% | 49% | | 13% | 45% | 58% | |
| Lithuania | 5% | 0% | 6% | 10% | | <1% | <1% | 1% | |
| Spain | 51% | 31% | 47% | 60% | | 7% | 27% | 36% | |
| Navarre (Spain) | 60% | 39% | 55% | 67% | | 7% | 27% | 36% | |

^a^ European Centre for Disease Prevention and Control (ECDC). Surveillance report on interim COVID-19 vaccination coverage in the EU/EEA, August 2024 – January 2025 [Internet]. 2024 [cited 2025 Apr 30].

**Figure S1. Patient exclusion flowchart, VEBIS hospital study, 19 September 2024–4 May 2025**

####
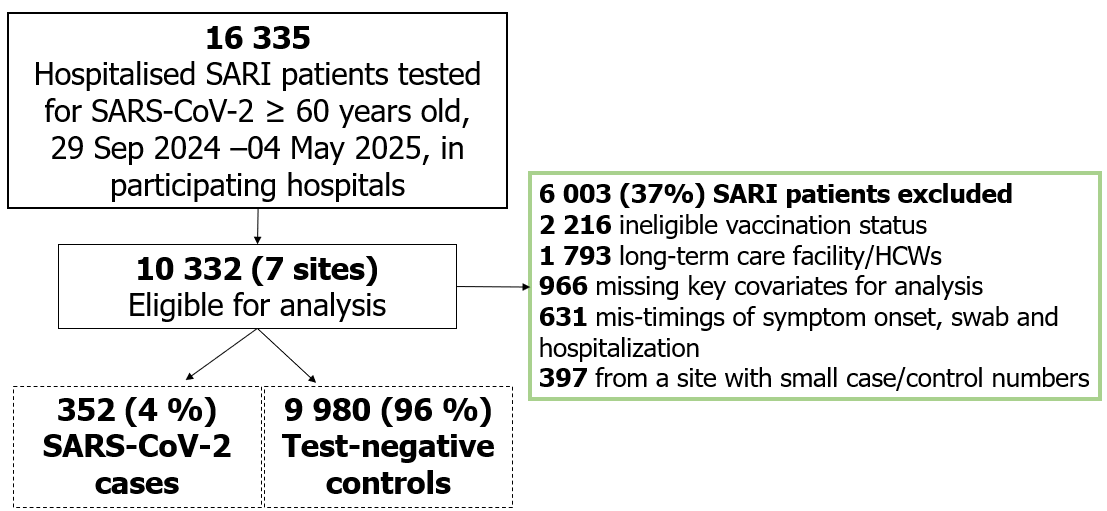


| **6 003 SARI patients excluded** |
| --- |
| **1 793 long-term care facility/HCWs/outside target group** |
| 1 165 residents in a long-term care facility, 6 healthcare workers and 622 outside the target group for vaccination |
| **966 missing key covariates for analysis** |
| 9 patients missing age, 8 missing sex and 949 missing information on common chronic diseases |
| **631 mistimings of symptom onset, swab and hospitalisation** |
| 427 patients swabbed > 10 days after symptom onset, 41 patients swabbed before symptom onset, 1 patient with symptom onset > 7 days after hospitalisation, 158 patients swabbed > 2 days after hospitalisation, 3 patients swabbed > 14 days before hospitalisation and 1 patient with symptom onset > 14 days before hospitalisation |
| **2 216 ineligible vaccination status** |
| 2 115 with last vaccine dose < 14 days before symptom onset, 57 with last vaccine dose received within 180 days prior to the campaign (unvaccinated), 9 with last vaccine dose received within 180 days prior to the campaign (vaccinated), 35 vaccinated with vaccine brand other than those approved by EMA |
| **397 patients from sites with small case/control numbers** |
| 313 patients were excluded because the total number of cases at the site was less than 5, and 84 patients were excluded because there were no vaccinated patients |
| **Records included are from 72 hospitals in 7 sites (BE, DE, ES, HU, IE, LT, NA).** |

#### Figure S2: Number of cases and controls by week of symptoms onset, VEBIS hospital study, Europe, 29 September 2024–4 May 2025 (n = 10 332)


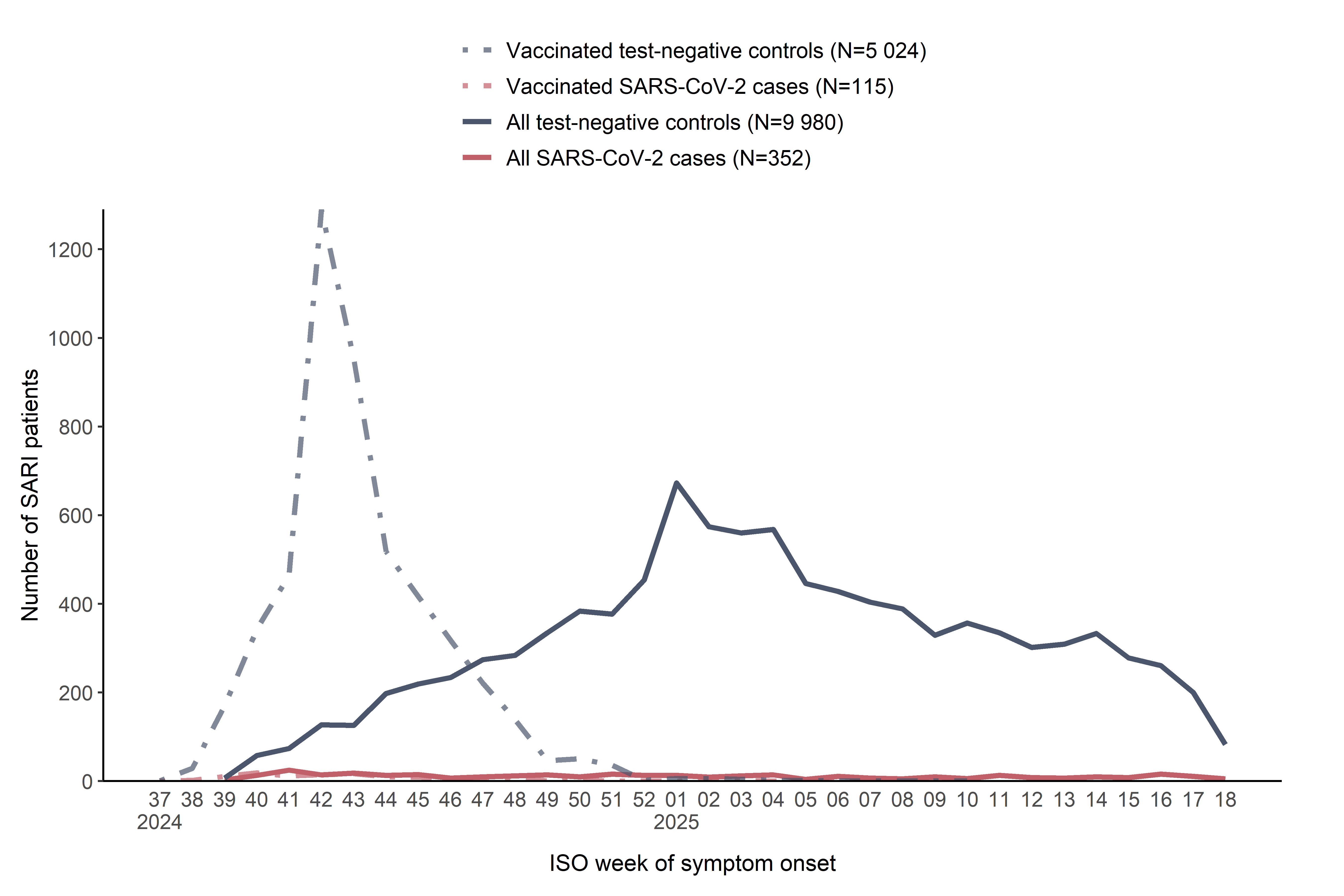


VEBIS: Vaccine Effectiveness, Burden and Impact Studies, SARI: severe acute respiratory infection, ISO: International Organization for Standardization

#### Figure S3: A) Number of COVID-19 cases by week of symptom onset by sublineage/lineage, B) Proportion of COVID-19 cases sequenced by week of symptoms, VEBIS hospital study, Europe, 29 September 2024–4 May 2024


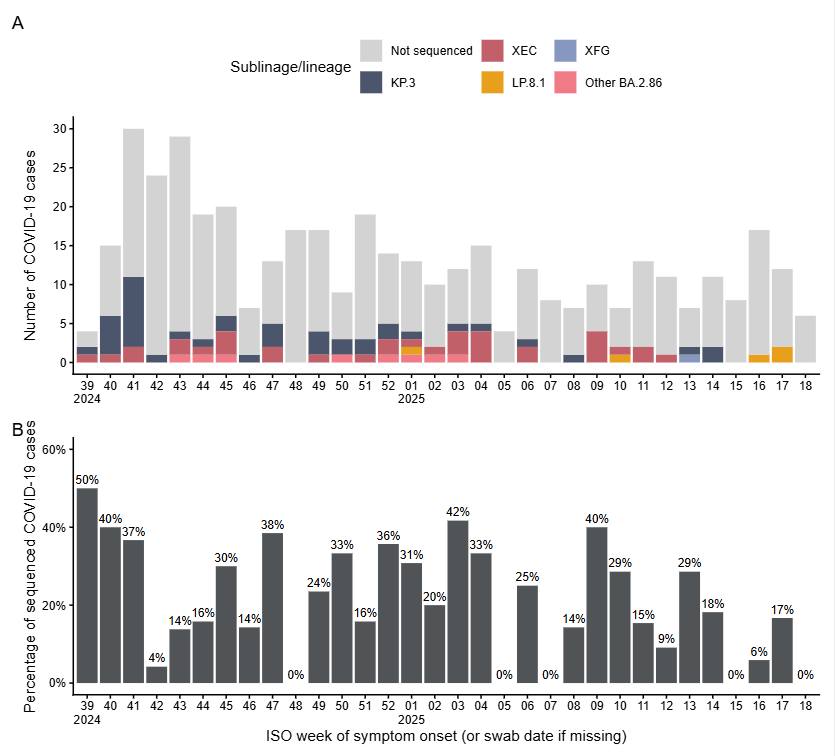


VEBIS: Vaccine Effectiveness, Burden and Impact Studies

There were 91 sequences included in the analysis. Omicron BA.2.86 lineages comprise: 42 (46%) KP.3, 35 (38%) XEC, 5 (5%) LP.8.1, 1 (1%) XFG, 8 other BA.2.86 (9%). Each participating country submitted sequences to the Global Initiative on Sharing All Influenza Data (GISAID) platform.

**Figure S4.** **Vaccine effectiveness of Comirnaty JN.1 vaccine against hospitalisation among SARI patients, by time since vaccination (60-day cut-offs), autumn 2024 vaccination campaign, VEBIS hospital study, Europe, 29 September 2024–4 May 2025 (n = 5 740)**


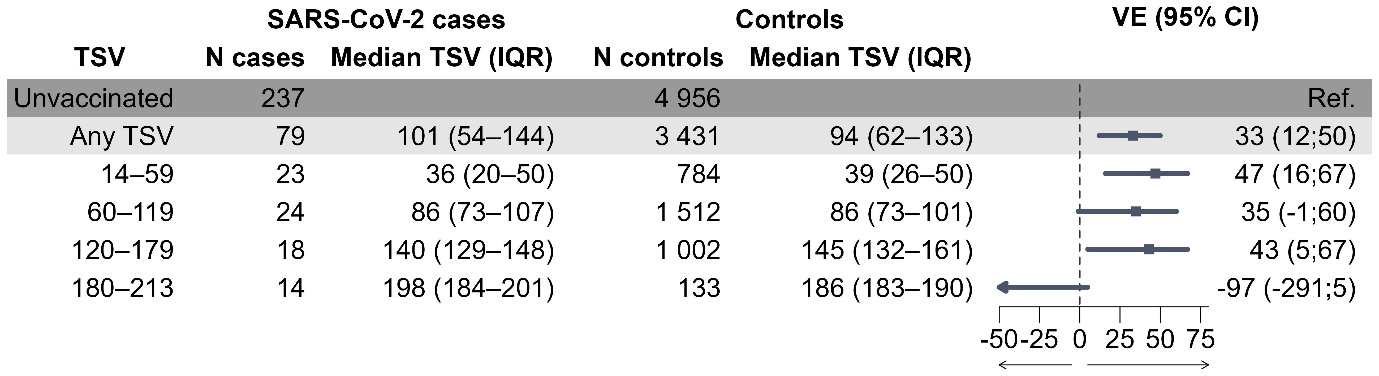


CI: confidence interval, IQR: interquartile range, TSV: time since vaccination (days from the last COVID-19 vaccination dose to symptom onset), VE: vaccine effectiveness, VEBIS: Vaccine Effectiveness, Burden and Impact Studies, dark grey shading: unvaccinated, light grey shading: any time since vaccination. Analysis including patients with available data on the Comirnaty JN.1 vaccine.
